# Mg^2+^ in the Major Groove Modulates B-DNA Structure and Dynamics

**DOI:** 10.1371/journal.pone.0041704

**Published:** 2012-07-23

**Authors:** Marc Guéroult, Olivier Boittin, Oliver Mauffret, Catherine Etchebest, Brigitte Hartmann

**Affiliations:** 1 Dynamique des Structures et Interactions des Macromolécules Biologiques, UMR 665 INSERM-Université Paris Diderot, Sorbonne Paris Cité, Institut National de la Transfusion Sanguine, Paris, France; 2 Laboratoire de Biochimie Théorique, UPR 9080 CNRS, Institut de Biologie Physico-Chimique, Paris, France; 3 Laboratoire de Biologie et Pharmacologie Appliquée, UMR 8113 CNRS-ENS de Cachan, Cachan, France; King's College, London, United Kingdom

## Abstract

This study investigates the effect of Mg^2+^ bound to the DNA major groove on DNA structure and dynamics. The analysis of a comprehensive dataset of B-DNA crystallographic structures shows that divalent cations are preferentially located in the DNA major groove where they interact with successive bases of (A/G)pG and the phosphate group of 5′-CpA or TpG. Based on this knowledge, molecular dynamics simulations were carried out on a DNA oligomer without or with Mg^2+^ close to an ApG step. These simulations showed that the hydrated Mg^2+^ forms a stable intra-strand cross-link between the two purines in solution. ApG generates an electrostatic potential in the major groove that is particularly attractive for cations; its intrinsic conformation is well-adapted to the formation of water-mediated hydrogen bonds with Mg^2+^. The binding of Mg^2+^ modulates the behavior of the 5′-neighboring step by increasing the BII (ε-ζ>0°) population of its phosphate group. Additional electrostatic interactions between the 5′-phosphate group and Mg^2+^ strengthen both the DNA-cation binding and the BII character of the 5′-step. Cation binding in the major groove may therefore locally influence the DNA conformational landscape, suggesting a possible avenue for better understanding how strong DNA distortions can be stabilized in protein-DNA complexes.

## Introduction

Specific DNA base-cation interactions have instigated numerous studies (reviewed in [1]–[3]) because it is presumed that cations can modulate DNA structure in a sequence-dependent manner, thus affecting the readout and packaging of DNA. Most of these studies are devoted to divalent cations because, in contrast to monovalent alkali cations, divalent cations can be unequivocally located in X-ray structures when they are ordered in the lattice [1]–[4]. In addition, DNA-cation interactions seem preferentially involve divalent cations – at least Mg^2+^ – over monovalent cations [5].

Within crystals, hydrated divalent cations frequently mediate intermolecular contacts between adjacent DNA molecules. Mg^2+^, Ca^2+^, or Mn^2+^ cross-link DNA bases, especially guanines, to phosphates of neighboring helices [6]–[14]. Molecular dynamics simulations have shown that Mg^2+^ stabilizes groove-backbone interactions in right-handed DNA crossovers [15]. Cations mediating intermolecular interactions thus behave as adhesives between helices and may play a biological role in crowded DNA media.

Both NMR and crystallographic approaches have been used to localize cations in DNA grooves where more specific intramolecular interactions link cations and groove atoms.

Despite potential steric hindrance due to the minor groove width, the minor groove is able to accommodate divalent cations. NMR experiments shows that divalent cations interact with the 5′-ApA minor groove floor in tracts of ApApApA [16], [17]. Mg^2+^ and Ca^2+^ inter-strand coordination modes have been observed at pure A:T sites [7], [11], [18] and, in some cases, at mixed A:T/G:C sites [7], [9], [13]. The strong electronegative potential of A:T minor groove is likely a key factor for constituting favorable cation-binding sites.

In the major groove, cations may preferentially bind to purines, in which O6/N7 guanine and N7 adenine atoms offer excellent anchoring points for ions [19], [20]. Various experimental results have shown that divalent cation interactions require dinucleotides containing at least one guanine [7], [21]. NMR spectra of a series of oligomers in presence of either paramagnetic Mn^2+^ and Co^2+^ or diamagnetic Zn^2+^ have shown H8 resonance broadening or shift for guanines in various sequence contexts [21]–[24]. The most pronounced effects occur on guanines in GpG or GpA and, to a lesser extent, guanines in GpT and GpC. These interesting results can be better interpreted in light of high-resolution X-ray DNA structures, which provide a detailed description of cation intra- and inter-strand cross-links. Intra-stand interactions seem to be predominant, with Ca^2+^ and Mg^2+^ coordinating the N7 and O6 atoms of GpG or ApG [7], [9] and, in one case, of GpA [11]. Less frequently, inter-strand bridges have also been observed, with Mg^2+^ coordinating the guanines of GpC•GpC at one end of the Dickerson-Drew dodecamer [13], [25] or G and T in GpA•TpC in a different sequence obtained at low temperature [14]. Fourier-transform infrared investigations on calf thymus DNA demonstrate that the interaction between Mg^2+^ or Ca^2+^ and the N7 of purines was not an artifact due to the short lengths of the oligomers studied by NMR and crystallography [26]. From all of these results, one can conclude that divalent cations within the major groove are able to bridge a guanine to an opposite base but preferentially form intra-strand bridges within G-containing dinucleotides.

Many studies therefore concur and have established that divalent cations interact with DNA bases within the grooves. However, the effect of cations on DNA structure is still unknown and is not a trivial issue to address. The main problem is to separate cause from effect. Does cation binding induce structural adjustments in DNA, or do cations recognize an intrinsic structural property in the sequence that favors their binding? Further, because our knowledge is mainly based on crystallographic structures, observed DNA distortions are difficult to interpret, as they may be due to lattice influences, the presence of inter-molecular contacts and, more generally, biases intrinsic to the solid-state approach. Thus, the impact of divalent ions buried in minor grooves on groove dimensions remains controversial [7], [9], [13], [25]. There are nonetheless several indications that DNA curvature is sensitive to divalent cations in the major groove. In crystals, DNA locally bends when Ca^2+^ or Mg^2+^ bridge the two guanines in GpG, compressing the major groove [7], or when Mg^2+^ binds opposite guanines in GpC•GpC [13], [27]. Some experiments in solution also support the possibility that divalent counterions influence DNA curvature, but these experiments could not specify their locations. Mg^2+^, Ca^2+^, or Zn^2+^ increase the gel mobility anomaly of GGGCCC repeats [28]. Also, Zn^2+^- or Mg^2+^- induced bends have been detected in G:C rich regions embedded in d(A)n tracts by atomic force microscopy [29] and NMR measurements of diffusion coefficients [30]. However, the molecular basis of such possible effects of divalent counterions on DNA remains far from well-characterized and understood.

The present work focuses on divalent cations located in the DNA major groove. We first analyzed divalent cation interactions in high-resolution X-ray DNA structures, using recent deposits in the Protein Data Bank. Upon identification of one major interaction pattern, extensive molecular dynamics (MD) simulations were then used to examine in solution the stability of this identified DNA-Mg^2+^ interaction and its impact on DNA structure and dynamics. Poisson-Boltzman calculations helped to build a credible scenario of DNA-Mg^2+^ interactions. These investigations were done using the so-called Jun-Fos oligomer, which has been exhaustively studied by NMR and modeling [31]–[34]. Possible repercussions of these findings involve DNA-protein interactions, particularly nucleosome structures in which numerous cations coordinate DNA.

## Results

### Divalent cations in the DNA major groove: analysis of X-ray structures

**Figure 1:**
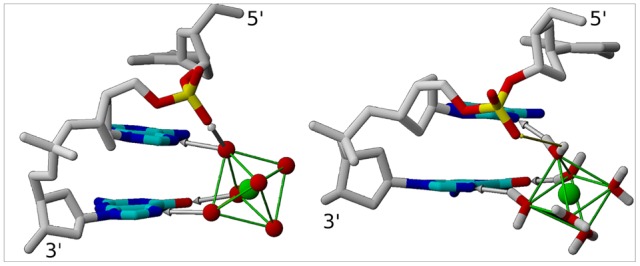
Interaction of Mg^2+^ in the DNA major groove. The 5′-CpApG-3′ fragment binds Mg^2+^ in both crystallographic (left) and simulated (right) structures. Mg^2+^ (green) forms water-mediated hydrogen bonds (gray arrows) with the N7 of both purines and the O6 of the guanine *via* its first hydration shell (left: oxygen atoms in red; right: explicit water molecules). The phosphate groups of CpA and ApG are in BII and BI conformation, respectively. Owing to the orientation of the O2P-O1P vector in the BII-CpA linkage, an additional hydrogen bond occurs between Mg^2+^ and O2P (left) in several X-ray structures. For the same reason, Mg^2+^ remains close to O2P in the simulated structures (right, thin yellow arrow).

The dataset of high resolution X-ray DNA structures (listed in Materials and Methods), including structures recently deposited in the PDB, provided for the examination a total of 23 major groove environments with divalent cations. In 20 cases, the DNA-cation interactions involve two successive purines of ApG or GpG steps (R_i–1_p_i_G_i_), confirming the predominance of intra-strand cross-links [7], [9]. Focusing our analysis on this frequent interaction pattern, we found that four Ca^2+^ and all of the 14 Mg^2+^ interact with the N7/O6 electronegative atoms of these bases *via* their first hydration shells (Figure 1). In two cases, Ca^2+^ directly coordinates the bases. These types of interaction have been previously described in detail (reviewed in [3]).

**Figure 2:**
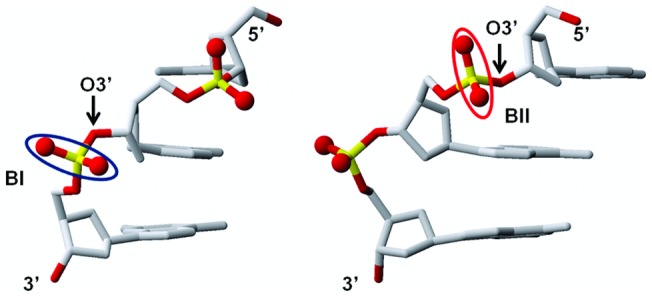
BI and BII phosphate groups . The two panels represent two views of the same 5′-CpApG-3′ fragment and highlight the structural differences between BI (p in GpA, circled in blue in the left panel) and BII (p in CpA, circled in red in the right panel) phosphate groups. The orientation of the O1P-O2P vector is roughly perpendicular and parallel to the axis of the double helix in BI and BII, respectively. The O3′ atom points outside the helix in BI whereas it is turned towards the helix center in BII.

Regarding DNA structure, we examined the two crucial inter-base pair parameters of twist and roll, and the conformational state of the phosphate groups (BI and BII conformers, represented Figure 2), three descriptors that are correlated in crystals as well as in solution [32], [34]–[41]. From this structural point of view, R_i–1_p_i_G_i_ interacting with ions are characterized by low twist (25.8±4.6° on average), positive roll (7.9±2.9° on average), and BI phosphate groups. Interestingly, the preceding steps, Cp_i–1_A_i–1_ or Tp_i–1_G_i–1_, also exhibit homogeneous structural features, with twist and roll of 49.3±4.7° and −9.8±2.8°, respectively; their phosphate linkages are in BII. These two contrasted profiles, low twist/positive roll/BI conformer and high twist/negative roll/BII conformer, illustrate the couplings imposed by the intrinsic mechanics of B-DNA [32], [34]–[41].

It is established that positive roll is required to allow divalent cation interaction with R_i–1_p_i_G_i_ sites [7]. Thus, cations indirectly impose the BI backbone state, disqualifying the BII state, which is incompatible with positive roll. This is manifest on the phosphate groups of GpG, all in BI in the presence of cations, although they are particularly conducive to BII [42]. The observation of BII-p_i–1_ is more intriguing, especially for TpG that shows a non negligible but yet moderate BII propensity [42]. This feature can be better understood by considering the structural implications of the BI→BII transition and its relationship with cations. The BII backbone conformation differs from its canonical BI alternative in regard to the torsion angles ε and ζ, which are respectively *trans/g-* in BI (ε-ζ ∼ −90°) and *g-/trans* in BII (ε-ζ ∼ +90°) [37]. This ε/ζ crankshaft induces two major structural changes in the phosphate group (Figure 2): first, the orientation of the O1P-O2P vector is roughly perpendicular and parallel to the axis of the double helix in BI and BII, respectively; second, the O3′ atom that points outside the helix in BI is turned towards the center in BII. In particular, the phosphate group motion from BI to BII shortens the distance between O2P(p_i–1_) and N7(R_i–1_) by roughly 1.8 Å. Therefore, any ion binding to N7(R_i–1_) will be closer to O2P(p_i–1_) when p_i–1_ is in BII. In nine occurrences of our dataset, hydrogen bonds were observed between O2P(p_i–1_) and one water molecule belonging to the cation hydration layer (Figure 1). In the absence of visible or stable hydrogen bonds (11 cases), the short distances (5.8±0.2 Å on average) separating O2P and O3′ from cations implies the existence of significant electrostatic interactions.

This analysis of crystallographic data confirms the previous observation that divalent cations in the major groove preferentially cross-link dinucleotide steps containing at least one guanine. These steps are characterized by low twist, positive roll and a BI backbone state. However, this analysis also suggests that cations influence the structure and dynamics of the 5′-step by interacting with its phosphate group. This was further explored using theoretical modeling approaches.

### Overview of the simulations

Molecular dynamics (MD) simulations in explicit solvent were done on the Jun-Fos oligomer. In addition to benefiting from an experimental support [31]–[34], this 14 bp oligomer contains a motif, CpApG, which appears to be recurrently contacted by cations in our X-ray dataset.

Four simulations of the Jun-Fos oligomer were considered (Table 1, detailed in Materials and Methods). The RMSD values calculated between canonical B-DNA and the simulated structures were very stable during all simulations. The structural characteristics of B-DNA such as χ values and sugar conformation were realistic. Convergence of the MD simulations with respect to the DNA structure was checked by comparing S1–S3. Statistics for the DNA descriptors give very similar results when extracted from any of these three trajectories. For instance, the average twist values of A_22_pG_23_ -which binds Mg^2+^- are 28.7±4.4°, 28.7±4.2° and 29.2±3.9° in S1, S2 and S3, respectively. Also, the phosphate group equilibrium, which is crucial for our study, is well reproduced through the trajectories, with between 36 and 40% of BII conformers on C_21_pA_22_ in S1–S3.

**Table 1:** MD simulations performed on the Jun-Fos oligomer.

Name	Ions	Restraints on Mg^2+^	t_total_ (ns)	t_int_ (ns)	ff for Mg^2+^
S0	Na^+^	N.A.	50	N.A.	N.A
S1	Na^+^ Mg^2+^	No	50	20	Amber default
S2	Na^+^ Mg^2+^	No	25	18.5	[15]
S3	Na^+^ Mg^2+^	Yes	75	75	Amber default

The simulations were performed with solvent containing Na^+^ or Na^+^ and Mg^2+^. Mg^2+^ was either free of restraints or restrained to be close (<5.5 Å) to A_22_ and G_23_. t_total_ is the total duration of the trajectories. For S1–S3, t_int_ is the duration of the interaction between Mg^2+^ in the major groove of A_22_ and G_23_. Two force field parameters for Mg^2+^ (ff for Mg^2+^) were used. N.A.: not applicable.

### Mg^2+^ binding in the DNA major groove: effect on DNA structure and dynamics

**Figure 3:**
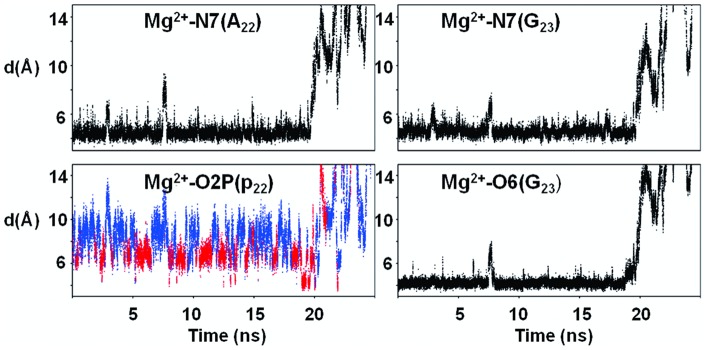
Distances characterizing the interaction between Mg^2+^ and DNA during the MD simulations. The distances between Mg^2+^ and the electronegative atoms N7(A_22_ and G_23_), O6(G_23_) and O2P(p_22_) belonging to the p_22_A_22_p_23_G_23_ fragment were extracted from S1. Mg^2+^ interacts with A_22_ and G_23_ *via* its first hydration shell for roughly 20 ns, as shown by the values of the distances d(Mg^2+^ – N7(A_22_)), d(Mg^2+^ – N7(G_23_)), and d(Mg^2+^ – O6(G_23_)). O2P(p_22_) is transitorily close to Mg^2+^. Short Mg^2+^ – O2P(A_22_) distances are associated with p_22_ in BII (BI in blue, BII in red). Identical interaction patterns are observed in S2 and S3.

We first present the results for S1 and S2, in which Mg^2+^ is free to escape from its initial position, close to A_22_ and G_23_. Mg^2+^/DNA interactions, monitored by the distances between Mg^2+^ and the electronegative atoms of A_22_ and G_23_ (example from S1: Figure 3), are observed during 20 and 18.5 ns in S1 and S2, respectively. These similar results show that the cation force field parameters do not significantly impact the Mg^2+^ behavior, since S1 and S2 use different parameters for the cation, either from Amber (S2) or from Varnai and Timsit [15]. During the time where Mg^2+^ interact with the oligomer, the low root mean square fluctuation (RMSF) values of Mg^2+^ (1.55 Å^2^ on average) reflect the existence of a well-defined attractive cation pocket. In the simulated structures, three water molecules of the Mg^2+^ hexahydrate cluster form hydrogen bonds with N7(A_22_, G_23_) and O6(G_23_), as observed in the crystallographic structures (Figure 1). The residence times of Mg^2+^ in the major groove indicates that B-DNA is likely not a Mg2+ binder as strong as RNA [43], [44], for instance the major groove of G•U wobble pairs [45]. However, the observation of ∼20 ns DNA-Mg^2+^ interactions ensures that the X-ray pattern is not an artifact, and that B-DNA in solution can transiently capture Mg^2+^.

The Mg^2+^/DNA interactions extracted from S1–S3 present identical characteristics. Thus, the snapshots of the trajectories S0–S3 (Table 1) were sorted in two groups. The first one contains the structures in which Mg^2+^ does not interact with the DNA major groove, for a total duration of 86.5 ns. The second group includes the snapshots characterized by water mediated hydrogen bonds between Mg^2+^, A_22_ and G_23_, which cover 113.5 ns.

**Figure 4:**
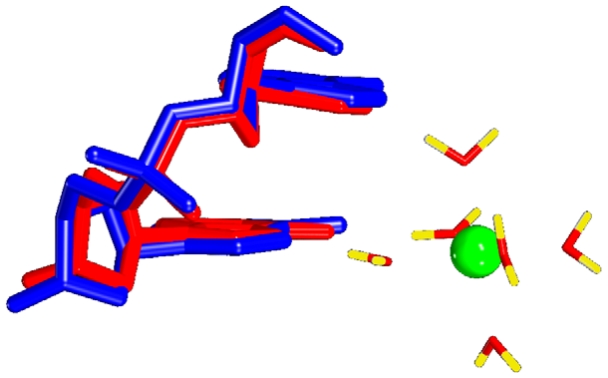
ApG step with and without Mg^2+^. Superimposition of the average structures of A_22_pG_23_ either free of Mg^2+^ (blue) or binding Mg^2+^(red). In both cases, this step shows the same structural characteristics (low twist, positive roll and BI phosphate group). ApG is thus ideally adapted to the octahedral structure of hydrated Mg^2+^.

Independently of the presence of Mg^2+^, A_22_pG_23_ is characterized by low twist (28.8±4° on average) and positive roll (4.6±4° on average). p_23_ is confined in BI, with less than 4% of BII. Indeed, ApG is not conducive to BII [42]. These ApG structural features are ideally adapted to the octahedral structure of hydrated Mg^2+^ (Figure 4). The distance between the two N7 atoms of A_22_ and G_23_ is 3.5±0.2 Å on average, allowing the bridging of two water molecules belonging to the first solvation shell of Mg^2+^. The other conformational profile encountered in B-DNA, *i.e.* high twist/negative roll/BII phosphate, pushes the two N7 atoms away (N7–N7 distance of 4.2±0.2 Å on average) and is thus less convenient for binding Mg^2+^. Our simulations confirm that bridging two successive purines by Mg^2+^ necessitates low twist and positive roll [7] and, indirectly, BI phosphate group.

**Figure 5:**
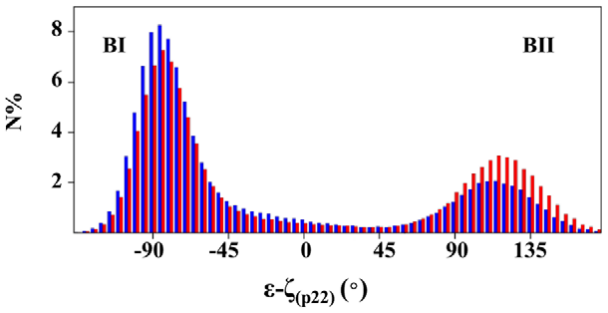
(ε-ζ) distribution without and with Mg^2+^ in the DNA major groove. N is the percentage of snapshots within each (ε-ζ)_p22_ interval. The distribution of (ε-ζ)_p22_ values was calculated on two groups of structures extracted from S0–S3. The first group (blue) corresponds to snapshots in which Mg^2+^ does not interact with the DNA major groove. In the second group (red), Mg^2+^ binds to the N7 and O6 atoms of A_22_/G_23_ through three water-mediated hydrogen bonds. The BII population of the 5′ phosphate group, p_22_, increases.

**Figure 6:**
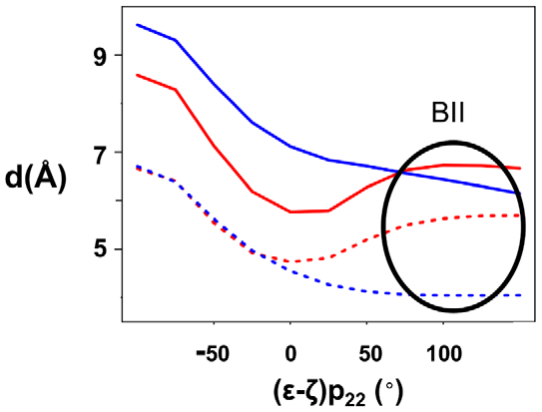
Effect of p_22_ conformational state on Mg^2+^-p_22_A_22_ distances. The distances between the OP2 and O3′ atoms of p_22_ and A_22_ (d(O2P(p_22_)-N7(A_22_)), indicated by a dashed red line; (d(O3′ (p_22_)-N7(A_22_) by a dashed blue line) depend on the conformational state of p_22_, represented here by (ε-ζ) p_22_ values. When Mg^2+^ interacts with A_22_ and G_23_, d(Mg^2+^-O2P(p_22_)) (red) and d(Mg^2+^-O3′ (p_22_)) (blue) are parallel to d(O2P(p_22_)-N7(A_22_)) and d(O3′ (p_22_)-N7(A_22_), respectively. The standard deviations for distances are 1.2 Å. The distances were examined on the two groups of structures defined in Figure 5 caption.

In contrast with p_23_, p_22_ undergoes BI↔BII transitions. C_21_p_22_A_22_p_23_G_23_ with p_23_ in BI and p_22_ either in BI or BII differ by ∼1 Å of RMSD. BI/BII populations of p_22_ are modulated by Mg^2+^ (Figure 5). In absence of Mg^2+^, p_22_ presents on average 24% of BII conformers ((ε-ζ) >0°). This percentage reaches 39% when hydrated Mg^2+^ coordinates A_22_ and G_23_. Thus, Mg^2+^ interacting with A_22_ and G_23_ increases the proportion of the p_22_ BII population. This latter result and the observations made on the crystallographic structures prompted a careful examination of the spatial relationship between Mg^2+^ and the atoms belonging to p_22_. The distances between O2P/O3′ (p_22_) and N7(A_22_) depend on the conformational state of p_22_ (Figure 6) owing to the structural changes during the BI↔BII transition, as mentioned above (Figure 2). When Mg^2+^ interacts with A_22_, both d(O2P(p_22_)-Mg^2+^) and d(O3′ (p_22_)-Mg^2+^(A_22_)) are shortened for stable BII conformers ((ε-ζ) >75°) (Figure 6). These distances are compatible with significant electrostatic interactions between Mg^2+^, O3′ (p_22_) and O2P(p_22_) but remain generally too great to allow the formation of additional hydrogen bonds. In particular, Mg^2+^-O2P water-mediated hydrogen bonds are observed in nearly the half of the crystallographic interactions whereas they were only marginal in our simulations (3.5% of the DNA-Mg^2+^ interactions). Actually, the Mg^2+^ locations in X-ray and simulated structures differ slightly (Figure 1). Mg^2+^ remains coplanar with the guanine during the trajectories, a position that increases the distance between the cation and O2P compared to the X-ray structures. Without excluding a possible force field bias, we cannot reasonably expect that Mg^2+^ locations are exactly identical in the solid state (*i.e.* from the crystallographic dataset) and in solution (*i.e.* from simulations).

**Figure 7:**
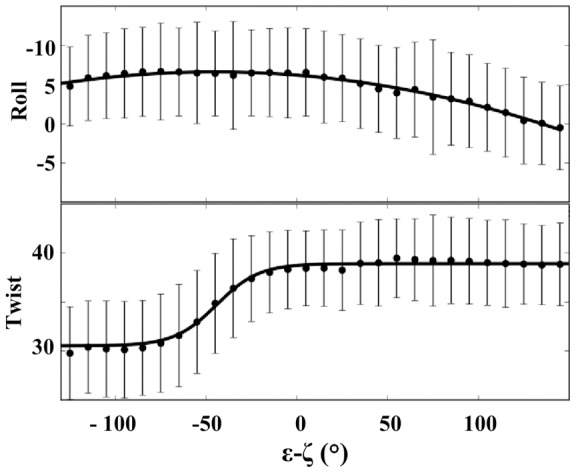
Changes in inter-base pair parameters as a function of (ε-ζ) values. The C_21_p_22_A_22_ average values of roll (°) and twist (°) and their standard deviations (bars) are plotted as a function of the (ε-ζ) values of p_22_, considering S0–S3. Mg^2+^-mediated enhancement of the BII population of p_22_ leads to an increase in the proportion of conformations characterized by negative rolls and high twists.

The twist and roll of C_21_pA_22_ cover a large range of values, positive/negative rolls and low/high twists being associated with BI and BII conformers, respectively, according to the intrinsic B-DNA mechanics. The evolution of twist and roll as a function of (ε-ζ), shown Figure 7, is common to all the simulations. A clear transition between low and high twist values occurs around (ε-ζ)  = −30°. Roll values change more slowly and are more gradual. Mg^2+^-mediated increase in the pure BII population of p_22_ leads to an increase in the proportion of the high twist/negative roll conformation in C_21_p_22_A_22_ without affecting the structural couplings characteristic of the double helix.

In sum, the interaction between Mg^2+^ and the DNA major groove involves the C_21_p_22_A_22_p_23_G_23_ fragment. The intrinsic structural properties of A_22_p_23_G_23_ match the octahedral configuration of the hydrated cation. Mg^2+^ modulates p_22_ behavior, favoring BII conformers. In turn, the conformational landscape explored by C_21_p_22_A_22_ is modified in terms of roll and twist. The proximity between Mg^2+^ and both O2P(p_22_) and O3′ (p_22_) indicates that p_22_ actively participates in the electrostatic component of the interaction, which is investigated in the next section.

### Electrostatic relationships between Mg^2+^ and DNA

Poisson-Boltzmann calculations were undertaken on a series of snapshots extracted from S1 to investigate the effects of the dinucleotidic sequence and structure on the DNA-Mg^2+^ interaction.

First, the electrostatic attractiveness of the DNA major groove for cations was examined along the 6 bp fragment T_20_pC_21_pA_22_pG_23_pA_24_pA_25_. We focused on a series of 0.5 Å sided cubes, calculating and averaging the electrostatic potentials of 9 points per cube (8 vertices and the center). These cubes were positioned close to each dinucleotide N_i–1_p_i_N_i_ so that they reproduced the average location of Mg^2+^ at A_22_pG_23_ in the simulations.

The electrostatic potential values of each N_i–1_p_i_N_i_ major groove region, sorted according the conformational state of p_i–1_, are summarized Table 2. The proximity between the electronegative atoms of p_i–1_ and N_i–1_ induced by p_i–1_ in BII tends to reinforce the electronegative character of the major groove. However, the electrostatic potentials are primarily subordinated to the dinucleotide sequence. Owing to their electronegative atoms in the major groove, G-containing dinucleotides, especially ApG, induce stronger negative potentials than ApA, CpA or TpC.

**Table 2:** Electrostatic potential values at cation locations in the DNA major groove.

p_i–1_N_i–1_p_i_N_i_	Ep (kT/e) for p_i–1_ in BI	Ep (kT/e) for p_i–1_ in BII
p_20_T_20_p_21_C_21_	−2.4 (0.6)	−2.2 (0.2)
p_21_C_21_p_22_A_22_	−2.7 (0.5)	−3.2 (0.6)
p_22_A_22_p_23_G_23_	−3.8 (0.7)	−4.3 (0.6)
p_23_G_23_p_24_A_24_	−3.3 (0.7)	−3.5 (0.5)
p_24_A_24_p_25_A_25_	−2.9 (0.4)	−3.2 (0.5)

Poisson-Boltzmann electrostatic potentials (Ep) were calculated for 0.5 Å sided cubes centered on the average position of Mg^2+^ observed in MD structures (Figure 1). These squares were shifted along the five N_i–1_p_i_N_i_ dinucleotide steps composing the T_20_p_21_C_21_p_22_A_22_p_23_G_23_p_24_A_24_p_25_A_25_ fragment. The potentials are sorted according to the conformational state of p_i–1_. A total of 100 snapshots extracted from S1 were examined. Standard deviations are given in parentheses.

Additional Poisson-Boltzmann calculations were done to better define the region and the atoms the most exposed to the electrostatic effect of Mg^2+^ during its interaction with DNA. Electrostatic potentials were calculated on DNA atomic positions, upon either removing (Ep_ref_) or keeping Mg^2+^ (Ep_Mg2+_). We consider here the difference between these two values, ΔEp  =  Ep_ref_ – Ep_Mg2+_, and examined the different DNA components, *i.e.* the sugars, bases and phosphate groups (P, O1P, O2P, O3′ and O5′), along the sequence. As expected, important modifications in electrostatic potentials occur in the region surrounding the bases that bind the cation, A_22_ and G_23_. The results are summarized in Table 3. Mg^2+^ induces strong changes on the electrostatic potential of the electronegative atoms of A_22_ and G_23_. In addition, the A_22_ neighbor, A_24_, and the partners of A_24_ and A_22_, T_5_ and T_7_, also feel the presence of the cation. The electrostatic potentials of neither the sugar nor the phosphate groups are affected by Mg^2+^, apart from the noticeable case of p_22_. The electrostatic changes on p_22_, especially on O2P and O3′, are particularly marked when p_22_ adopts the BII conformation. According to the MD simulations, this electrostatic effect is not sufficient to completely trap p_22_ in BII. However, it explains the shift of the BI↔BII equilibrium towards BII. Therefore, N7(A_22_), N7(G_23_), O2P(p_22_) and O3′ (p_22_) are major players in the interaction of Mg^2+^ within the DNA major groove.

**Table 3:** Effect of Mg^2+^ on DNA atomic electrostatic potentials around the cation binding site.

DNA atom or group of atom	ΔEp (kT/e) for p_22_ in BI	ΔEp (kT/e) for p_22_ in BII
O4(T_7_)	−1.3 (0.3)	−1.5 (0.3)
O4(T_5_)	−2.1 (0.2)	−2.1 (0.3)
**N7(A_22_)**	−3.1 (0.2)	−3.2 (0.3)
**N7(G_23_)**	−2.2 (0.3)	−2.3 (0.3)
**O6(G_23_)**	−0.9 (0.3)	−1.9 (0.5)
N7(A_24_)	−1.7 (0.3)	−1.7 (0.3)
p_22_	−3.9 (0.3)	−8.3 (0.3)
O3′ (p_22_)	−0.6 (0.2)	−2.1 (0.3)
O2P(p_22_)	−1.1 (0.3)	−2.2 (0.5)

ΔEp represents the electrostatic effect of Mg^2+^ on various DNA atoms or groups of atoms. Mg^2+^ binds to A_22_ and G_23_ (in bold) in the C_21_p_22_ **A_22_**p_23_ **G_23_**p_24_A_24_ fragment. ΔEp values were calculated for p_22_ either in BI (ε-ζ<−50°) or BII (ε-ζ>75°). T_7_ and T_5_ are the partners of A_22_ and A_24_, respectively. The phosphate group p_22_ is comprised of six atoms, O3′, O1P, P, O2P and O5′. A total of 90 snapshots extracted from S1 were examined. Standard deviations are given in parentheses.

These calculations create a credible scenario for Mg^2+^ interacting with the DNA major groove. Hydrated Mg^2+^ takes advantage of the negative pocket in the major groove of two successive bases, one guanine and one purine, to bridge them by water-mediated hydrogen bonds. When the 5′ phosphate group spontaneously shifts towards BII, its O2P and O3′ atoms move closer to the bound Mg^2+^, increasing their mutual electrostatic attractiveness. This additional interaction therefore helps stabilize the cation within the major groove and increases the BII population of p_i–1_.

## Discussion

Instigated by a body of crystallographic evidence, our modeling investigations show that Mg^2+^ is able to form stable intra-strand cross-links in the major groove of the ApG step in solution. The interaction network involves the first hydration shell of Mg^2+^ and the p_i–1_A_i–1_p_i_G_i_ fragment. A_i–1_p_i_G_i_ adopts a conformation characterized by positive roll and BI phosphate linkages. Our molecular dynamics approach demonstrates that the counterion increases the percentage of BII conformers of p_i–1_, such that Cp_i–1_A_i–1_ more frequently explores negative roll conformations. MD trajectories associated with Poisson-Boltzmann calculations helped reveal the mechanism underpinning the interaction of divalent cations with the DNA major groove. Cations are attracted to the major groove of A_i–1_p_i_G_i_, which is structurally well-adapted to cation binding and forms a strong and well-defined electronegative pocket. Both O2P and O3′ atoms of p_i–1_ benefit from a favorable electrostatic interaction with the cation, further enhanced when p_i–1_ is in BII. This latter interaction helps stabilize both the cation and BII conformers of p_i–1_.

Divalent cations bridging two successive guanines in the major groove have been suspected to induce local bends in X-ray structures [7]. Such cation-induced curvature is supported by indirect indications gleaned from observations in solution made on DNAs containing (G)_n_ tracts [28]–[30]. The Jun-Fos oligomer in the present study bends towards the major groove with an average curvature of 16° [34]. Our observations here show that this bending is not significantly affected by one bound Mg^2+^. Nevertheless, from a general point of view, curvature intensity depends on the succession of roll signs along the sequence, which are correlated with BI/BII conformations. Accordingly, it cannot be ruled out that Mg^2+^ influences the global curvature of particular sequences, for instance in (G)_n_ tracts, by accumulating modulations of their BI/BII ratio.

From a structural point of view, CpApG appears to form a very favorable cation binding site. In this trinucleotide fragment, an intrinsically BII-conducive phosphate group in CpA precedes an ApG step, ready to coordinate a hydrated cation. Our analysis of crystallographic structures reveals that a TpGpG tract is also an attractive site, likely because of the non negligible BII propensity of TpG and the strength of the electrostatic potential in the GpG major groove. Other trinucleotides, made of BII-rich dinucleotides followed by ApG, GpG or GpA, may also fix cations. In this line, another caveat concerns the balance between the Mg^2+^ residence times near the charged phosphate groups and near the major groove electronegative atoms. This point was previously addressed by a modeling study [46], which demonstrated that Mg^2+^ were more frequently found near phosphates than in the major groove. Yet, this study also suggested that the presence of Mg^2+^ in the major groove affects the bending occurring at A-tract flanking sequences. Given this context, systematic studies should to be done to inventory the most propitious sites for harboring cations.

Are our findings applicable to cations other than Mg^2+^? The examination of the six Ca^2+^ present in the crystallographic dataset strongly suggests that this physiological cation behaves similarly to Mg^2+^. Nevertheless, the stability of Ca^2+^ in the DNA major groove remains to be confirmed, given that its first solvation shell, crucial for the interaction, may be more labile than the Mg^2+^ solvation shell [9]. Regarding monovalent cations, ^31^P NMR results [33] show the differential influence of Na^+^ and K^+^ on the BI↔BII equilibrium of several phosphate linkages, unambiguously demonstrating that DNA in solution is influenced by the nature of the monovalent cation. The examination of the two crystallographic structures that contain bound monovalent cations in the major groove (Rb^+^, PDB code 3GG; Tl^+^, PDB code 3GGI) reveals an interaction scheme identical to the one observed here for divalent cations. We carried out several MD simulations not presented here, replacing Mg^2+^ with K^+^. Nevertheless, the residence times of K^+^ at the ApG step were very limited (at best 1.5 ns) compared to those observed with Mg^2+^. Such instability, also noticed in other DNA sequences [47], [48], hinders the acquisition of significant statistics on ion coordination and structural effects. Thus, the mechanism by which monovalent cations influence DNA behavior remains an open question.

Although divalent cations do not radically change DNA structure and dynamics, they appear to modulate DNA behavior and they may thereby help stabilize sharp DNA deformations in DNA-protein complexes. Ions in DNA wrapped around a histone core offer a good system through which the potential role of cations in DNA-protein interactions could be addressed. The nucleosome core particle (NCP) consists of 146–147 bp of DNA wound twice around an octameric core of four histone proteins. This DNA superhelical path induces a periodic alternation of positive and negative roll tracts [49], mainly associated with BI and BII phosphates, respectively [42]. In the best resolved NCP structure (PDB code 1KX5), 12 Mn^2+^ (mimicking Mg^2+^) are observed in the DNA major groove [50], [51]. Two of them form inter-strand bridges. The 10 remaining cations coordinate GpG or ApG, characterized by positive rolls and BI phosphates. The 5′-TpG or CpA neighbors of these cation-binding dinucleotides exhibit marked negative rolls (-13±8° on average) associated with BII phosphates, and their O2P atoms are involved in a water-mediated hydrogen bond with Mn^2+^. These features are surprisingly similar to those observed on free DNA. Hence, divalent cations may fix particular DNA conformations required by interacting proteins, limiting the energetic and entropic costs of protein binding.

In conclusion, the present study suggests that DNA structure and dynamics are sensitive to divalent cations in the major groove. Cation binding is sequence-dependent and modulates the intrinsic sequence-dependent properties of DNA in terms of the population of conformational states, including the BI↔BII equilibrium of phosphate groups. Although the role of cation-mediated modulation in DNA packaging and readout is largely speculative at this stage, it is an exciting hypothesis to test in regard to DNA-protein processes.

## Materials and Methods

### Crystallographic dataset

DNA crystal structures were selected based on the following two criteria: high or very high resolution and the presence of divalent cations in the DNA major groove. The dataset comprises 11 B-DNA decamers with a resolution of ≤1.65 Å (PDB codes: 3GGI, 3GGK, 3GGB, 1ZF7, 1ZFB, 1D23, 1ENE, 1EN9, 1EN8, 1EN3, 1D8G).

### DNA sequence

The double-stranded DNA oligomer studied using molecular dynamics simulations has the following 14 bp sequence: 5′-d(G_1_ C_2_ A_3_ T_4_ T_5_ C_6_ T_7_ G_8_ A_9_ G_10_ T_11_ C_12_ A_13_ G_14_ )-3′•5′-d(C_15_ T_16_ G_17_ A_18_ C_19_ T_20_ C_21_ A_22_ G_23_ A_24_ A_25_ T_26_ G_27_ C_28_ )-3′. This oligomer, called the Jun-Fos oligomer, has been exhaustively characterized by NMR and modeling [31]–[34].

### Molecular dynamics (MD) simulations

All simulations were performed using the AMBER 10 program [52], with the Parm98 force field [53] and under NMR restraints. Actually, in absence of experimental restraints, notorious shortcomings occur with AMBER force fields, especially regarding the backbone behavior [34]. Parm98 does not implement realistic BI↔BII equilibrium (related to ε-ζ) [34] and drives α/γ angles in unusual conformations [54]. Parmbsc0 [55] overly suppresses BII conformers [34] but improves the α/γ behavior, without totally avoiding undesirable flips [56]. Unfortunately, NMR restraints associated to Parmbsc0 do not rescue BI/BII populations [34]. In contrast, the same restraints used in conjunction with Parm98 restitute more acceptable behaviors for both ε-ζ and α/γ [31], [34]. It is the reason why we chose Parm98, supplemented by NMR restraints.

The starting point was a canonical B-DNA, neutralized with either 26 Na^+^ or 24 Na^+^ and one Mg^2+^ and hydrated with TIP3P [57] water molecules in a truncated octahedron simulation box. The distance between the center of DNA periodic images is ∼90 Å, which allows for a solvent shell extending at least 15 Å around DNA. Simulations were performed at constant temperature (300 K) and pressure (1 bar) using a Berendsen coupling algorithm [58]. The integration time step was 2 fs and covalent bonds involving hydrogen were constrained using SHAKE [59]. Long-range electrostatic interactions were treated using the particle mesh Ewald approach [60] with a 9 Å direct space cut-off and a 0.00001 Ewald convergence tolerance for the long range electrostatic interactions. The non-bonded pair-list was updated heuristically and the center-of-mass motion was removed every 10 ps.

Water molecules and cations were energy-minimized and equilibrated in the NVT ensemble at 100 K for 100 ps, with the DNA constrained. The entire system (DNA, water molecules and ions) was then heated from 100 to 300 K in 10 ps in 5 K increments with harmonic restraints of 5.0 kcal mol^−1^ Å^−2^ on the solute atoms. The simulations were continued in the NPT ensemble, without a noticeable change in volume. The positional restraints were gradually removed over 250 ps and followed by the production phases.

All MD simulations were run with a set of restraints consisting in three NMR internucleotide distances per dinucleotide, H2′_i_-H6/8_i+1_, H2″_i_-H6/8_i+1_, and H6/8_i_-H6/8_i+1_, inferred from ^31^P chemical shifts [31], [32]. These restraints were applied instantaneously via a mixed parabolic (for distance −10%) and hyperbolic (for distance +10%) potential with a force constant of 10 Kcal.mol^−1^.Å^−2^ around a central, flat-bottomed shape covering the experimental range of distances (estimated at 10% of the distances). We previously showed that this protocol best reproduces the exhaustive NMR data measured on the studied oligomer, while preserving the DNA dynamics [31]. Removing these NMR restraints in an additional 25 ns trajectory ensured that the main conclusions presented here were robust.

Four trajectories were considered (Table 1). S0, done in presence of Na^+^ counterions, will be the reference simulation. Three simulations, S1–S3, were carried out with Na^+^ and Mg^2+^, keeping the same starting point but reinitializing velocities. Mg^2+^ was initially restrained close to two bases of the Jun-Fos oligomer, A_22_ and G_23_, by three distance restraints (d(Mg^2+^ – N7(A_22_)) <4.8 Å, d(Mg^2+^ -N7(G_23_)) <6.0 Å and d(Mg^2+^ -O6(G_23_)) <5.0 Å), according to the preferred major groove location observed in X-ray DNA structures. In S1 and S2, the restraints were then gradually removed before running trajectories in which Mg^2+^ was thus free to escape from its initial position. To ensure robust statistics on DNA-ion interactions, an additional trajectory, S3, was carried out maintaining the restraints. The analysis covered a total of 200 ns of production runs.

The default Mg^2+^ parameters provided in AMBER were used in S1 and S3. S2 was carried out using Mg^2+^ parameters given by a recent study [15]. These parameters (r* = 0.6245 Å and ε = 28.4444 kcal mol^−1^) reinforce the van der Waals potential, and accurately reproduce the free energy of Mg^2+^ hydration and the radial distribution function of water.

### Electrostatic Calculations

Electrostatic potential maps were calculated with the Adaptive Poisson-Boltzmann Solver (APBS) [61] using a nonlinear solution. These calculations were done on snapshots extracted from trajectories, using APBS default parameters (physiological NaCl concentration of 150 mM, temperature of 298 K, solvent dielectric of 78.4, and solute dielectric of 2). Van der Waals radii and partial charges for DNA were those of the Parm98 force field. Solute charges were distributed onto grid points using a cubic B-spline discretization. The molecular surface was defined by the interface between a 1.4 Å solvent probe, corresponding to the radius of a water molecule, and the solute van der Waals radii.

### Structure analysis

X-ray and MD DNA structures were analyzed using the Curves+ algorithm [62]. The phosphate conformations were analyzed in terms of BI and BII states, defined by the value of the pseudo-angle (ε-ζ): BI corresponds to (ε-ζ) <0° and BII to (ε-ζ) >0°. The alternative atomic positions proposed in the PDB files of some X-ray structures (PDB codes: 3GGI, 3GGK, 3GGB, 1EN3, 1D8G, 1EN9) were analyzed separately. The first nanosecond of each MD was discarded from the analysis.

For clarity, we consider that p_i_ phosphate linkage is composed of the atoms O3′, O1P, P, O2P and O5′, noted X(p_i_).
